# Hedonic Risk Preference Associated with High-Risk Behaviors under COVID-19 Pandemic among Medical Students in Japan

**DOI:** 10.3390/ijerph20126090

**Published:** 2023-06-09

**Authors:** Zechen Zeng, Nobutoshi Nawa, Chie Hirama, Takeo Fujiwara

**Affiliations:** 1 Department of Global Health Promotion, Tokyo Medical and Dental University, 1-5-45 Yushima, Bunkyo-ku, Tokyo 113-8510, Japan; 2 Department of Medical Education Research and Development, Tokyo Medical and Dental University, 1-5-45 Yushima, Bunkyo-ku, Tokyo 113-8510, Japan

**Keywords:** risk preference, general risk preference, context-specific risk preference, COVID-19, risk behavior, youth, Japan

## Abstract

Background: Public health measures to mitigate the spread of COVID-19 have focused on raising awareness and disseminating knowledge. Few considered people’s risk preferences and no measurement was adapted to the context of COVID-19. This study aims (1) to investigate the association between risk preference and risk behaviors and (2) to compare a novel hedonic preference question with traditional risk preference assessment tools in the context of the COVID-19 pandemic among medical students in Japan. Methods: An online survey of fourth-year medical students was conducted. Logistic regression analysis adjusted for gender, age, household income, and the overconfidence effect were performed to investigate the association. Results: We observed significantly higher odds of high-risk behaviors in general risk preference (odds ratio (OR): 4.04; 95% confidence interval (CI): 1.05–15.50) and hedonic preference (OR: 6.58; 95% CI: 1.86–23.28) when adjusted, whereas monetary preference showed no significant association. Concerning specific risky behaviors, hedonic preference were significantly associated with four items after adjusting for covariates (“dine out” (OR: 2.78, 95% CI: 1.13–6.85), “go out” (OR: 4.35, 95% CI: 1.65–11.46), “not practicing safety precautions” (OR: 2.79, 95% CI: 1.11–7.04) and “travel” (OR: 4.36, 95% CI: 1.42–13.44)), and general preference in two (“dine out” (OR: 4.82, 95% CI: 1.66–14.00) and “go out” (OR: 6.48, 95% CI: 2.07–20.24)). Conclusion: Hedonic and general risk preferences were significantly associated with high-risk behaviors during the COVID-19 pandemic. Future application of the novel risk-for-pleasure-seeking preference question is warranted.

## 1. Introduction

The number of COVID-19 infections in Japan reached an unprecedented high during the fifth wave [1]. At the beginning of the COVID-19 pandemic, efforts were made to implement a “zero COVID” strategy [2], but as it turned out to be practically impossible and economically devastating to impose strict lockdowns, public health measures shifted to mitigating transmission by focusing on those considered to be the most socially active: young people. The mainstream media attributed the rising numbers to the general recklessness, ignorance, and overconfidence of the younger generation [3]. Think tanks for the Japanese government expressed deep concern about the noncompliance of young people. They proposed stricter restrictions on alcohol-consumption in restaurants (which usually includes talking closely without masks in confined spaces) on top of the existing awareness campaign [4]. Contrary to expectations, little effect of these measures was observed. Given the situation, it can be hypothesized that it was the willingness to take risks rather than ignorance or confidence that contributed more to risky behaviors among young people, as it is well documented that young people are more risk-seeking [5,6,7,8].

Risk preference is considered a vital part of the decision-making process, for it is associated with how people evaluate decisions and possible outcomes [9,10]. Empirical evidence demonstrates that risk preference is relatively stable over time, making it a reliable tool for predicting risky behaviors [11]. The basic concept of risk preference comes from the economic models of Expected Value and Expected Utility. Both models are based on the common assumption of economic rational choice theory [12]: people view risky options as distributions of possible outcomes. The value of each risky option is the aggregation of the product of the probability and the values of the possible outcomes, and the option with the higher total value is chosen [13]. In the theory, individuals are seen to be driven by the goals and desires that express their “preferences”. Given specific constraints and information, individuals will end up with the same action because they will always choose the alternative they believe will give them the greatest satisfaction or “highest utility” in economic terms [12]. It has become the normative model in the economic analysis of choice under risk that individuals’ constancy of intuitively choosing under the same set of conditions is required by maximizing the utility [14].

While rational choice theory has dominated decision-making theory in the economic domain, it has been further modified by psychological variables such as context and process [15]. Recent research shows that risk-taking decisions depend on context and process [16,17]. It has become evident that changing the way questions are framed provides a different context or leads people to a different decision-making process that may result in different choices [13]. In other words, context- and process-specific risk preference that resembles real-life risk-taking is optimal for predicting future risk behaviors. Therefore, risk preference assessment tools must be context- and process-sensitive to accurately reflect the complex decision-making process in real-life.

To date, there is no single best assessment tool that accounts for all processes and contexts. Especially in the context of the COVID-19 pandemic, where any action that is perfectly normal at any other time can be considered a risk behavior, no previous research has proposed a widely accepted model for categorizing or rationalizing risk behaviors in terms of risk preference. The literature on this topic has generally taken three approaches the measurement of risk preference: the self-reported general risk preference question, the expected utility-based lottery choice method, and the Domain-Specific Risk-Taking Scale (DOSPERT). 

Previous studies that have focused on the self-reported general risk preference question have found that it is associated with compliance with social distancing precautions [18,19]. The self-reported general risk preference (called “general preference” for short, hereafter) question is designed as an intentionally ambiguous question and is intended to measure participants’ self-assessment of overall stable risk preferences, which has been one of the most established forms of measuring risk preference with stable long-term results [20]. Other studies took the approach of the lottery choice method [21] and the DOSPERT Scale [22]. These approaches are very common in risk preference studies and typically predict various types of risky behaviors. While with the general risk preference question, we obtain a vague and general feedback of what the person deems as risky behaviors, with the approach of the lottery choice method and the DOSPERT Scale, we obtain specific risk propensity on specific aspects. However, these three assessment tools have been criticized for the lack of proper context and decision-making process. Such drawbacks make them alienated from real-life risk choices [5]. The COVID-19 pandemic has also drastically changed how we perceive risk. Therefore, a measurement tool that accommodates contexts and decision-making processes under the COVID-19 pandemic is needed.

Our monetary preference question is a simplified lottery choice method following the methods of Warneryd [23] within the framework of expected utility [24]. Then, we invented a similar question that altered the stakes from money to parties during the COVID-19 pandemic, which we refer to as “hedonic preference” for short, hereafter. The descriptive background of the COVID-19 pandemic provided the context, and we used the same lottery process that participants are now familiar with due to the monetary preference question. In order to replicate the decision-making process, we phrased the question in such a way that participants would only consider the risk–benefit trade-off of pleasurable events related to young people and ignore potential long-term health risks because (1) in real-life decision-making, people tend not to consider long-term risks and rely more on impulses [25], whereas when answering a questionnaire of questions related to COVID-19, long-term risk may be an influential factor and, (2) people tend not to answer honestly when ethical issues may be implied (in this case, contracting COVID-19). 

In summary, our goal was (1) to examine the association of three approaches to measuring risk preference—the self-reported general risk preference question, the expected utility-based lottery choice methods using the monetary preference question, and our original hedonic preference with risk behaviors in the COVID-19 pandemic—and (2) to compare our original hedonic preference question with traditional risk preference assessment tools (i.e., the self-reported general risk preference question and the expected utility-based lottery choice methods using the monetary preference question).

## 2. Methods

### 2.1. Participants and Data Collection

This study was part of the student survey during the public health course at Tokyo Medical and Dental University. To address the difficulty of separating knowledge from risk preferences [26], we chose fourth-year medical students as participants in this survey. The participants have completed all basic and clinical medical courses and have been regularly briefed by the school on updated COVID-19 information. Therefore, lack of sufficient understanding of COVID-19 was not expected to be a problem. At TMDU, fourth-year medical students would be assigned to a laboratory of their choice for a six-month research period before entering clinical clerkship in the fifth and sixth years. Little to no stress from exams, job hunting, or thinking about graduate school makes this the most carefree six months of the six years of medical school.

Past literature has identified several critical influencing factors, such as age, gender, socioeconomic status, education, cognitive abilities, and emotional state [5,27,28,29]. Traditionally, many of these studies, along with research in economic, social, and psychological disciplines, have predominantly utilized college students as subjects. This choice stems from the relative homogeneity of this group, particularly regarding age and educational level, which simplifies the research design and analysis while facilitating control over potential confounding factors. 

The questionnaire for all 108 fourth-year medical students was conducted via Google Form. We ensured that no personal information that could lead to specific individuals being identified was included. Students were given ample time to complete the questionnaire with a total of 120 questions. We added follow-up questions about the reasons for their choice in measuring risk preferences to ensure participants fully understood the choices they selected. The study was not incentivized for its voluntary and anonymous nature. Ethical review and approval were not required for the study.

### 2.2. Terminology

In our study, “risk-seeking” was referred to as both “risk-loving” and “relatively less risk avoidant”. We used “general preference”, “monetary preference”, and “hedonic preference” in short for “self-reported general risk preference”, “risk-for-money preference”, and “risk-for-pleasure-seeking preference”, respectively.

### 2.3. Measurements

#### 2.3.1. Risk Preference

Students were asked about their risk preferences based on three aspects: general, monetary, and hedonic. A brief summary of these three methods, based on the review of Charness et al. [20], is listed in the Supplementary Material (Tables S1 and S2).

General preference was assessed by the self-reported general risk preference question and was asked by, “Do you consider yourself a person who would take risks in general?”, which is well documented as the standard approach. We adopted the two-choice form of the Eysenck Personality Questionnaire [30] to avoid participants’ centralization in the middle. 

Monetary preference was assessed using the expected utility-based lottery choice methods. We used a simple lottery choice based on the expected utility theory [24]. The expected utility-based lottery method observes the “turning point” in a list of choices and assesses the so-called risk premium range of the two marginal choices respondents make. This method offers respondents choices of lotteries (i.e., a 50/50 chance between USD 2.00 and USD 1.60 or a 50/50 chance between USD 3.85 and USD 0.10 [21]). With the information of when a participant switches from one list to another, we can calculate the exponent θ from the utility as the function u(x) = x^θ^. The exponent θ indicates one’s risk preference. The risk preferences elicited through different approaches are rarely precisely the same [31], thus leaving us some degree of freedom in setting the stakes of the lottery. We created our monetary preference question following the methods of Warneryd [23]. 

Hedonic preference was assessed by the risk-for-pleasure-seeking preference question. The risk-for-pleasure-seeking preference question is a new measurement that we developed to target specific preferences related to pleasure-seeking during the COVID-19 pandemic. We changed the money stakes from the lottery method of our monetary preference to the hedonic stakes of parties, which we believed would resonate with young people. The numbers in the monetary preference and hedonic preference questions are the same for easy understanding. Since the COVID-19 pandemic was an emerging pandemic with no precedent, there was no previous risk preference research on how to properly reflect such context and process. It is also to be noted that the definition of “risky behaviors” drastically changed during the pandemic, which makes it difficult to compare the risk-for-pleasure-seeking preference question with other established methods. However, we held thorough discussions among public health experts to ensure the content validity of the hedonic preference.

The question is phrased as follows: 

“Suppose you were a famous YouTuber, and you love drinking with large groups of people. You are often invited to large drinking parties by your friends. However, there is a risk that the public will find out about it, and if they do, you will be suspended for a while and will not be able to go out drinking. You have been vaccinated and do not consider yourself at risk of infection. The only thing you care about in this world is the number of parties you can go to. What would you choose in the following situation?” and response items were:

1. Party 4 times a year with a 100% chance of not getting caught,
2. Party 5 times a year with an 80% chance of not getting caught, but a 20% chance of getting caught, leading to zero parties,
3. Party 8 times a year with a 50% chance of not getting caught, but a 50% chance of getting caught, leading to zero parties,
4. Party 10 times a year with a 50% chance of not getting caught, but a 50% chance of getting caught, leading to zero parties.

Follow-up questions about the reasons for participants’ choices were asked, and no signs of choosing without logical reasons were found (see Supplementary Material, Questionnaire S3 for the complete questionnaire).

#### 2.3.2. Risky Behaviors

There are five measuring items in this study: “travel”, “dining out”, “drinking at restaurants”, “going out with friends”, and “not practicing safety precautions”, which include “wearing masks”, “washing hands”, “taking the temperature”, and “avoiding crowds regularly”. All these behaviors were cautioned against by the Ministry of Health, Labour and Welfare of Japan [32]. Raising awareness of most of these behaviors was universal, and the logic behind it was easy to understand [33]. “Social gatherings with drinking alcohol” was explicitly listed because (1) drinking alcohol improves mood and decreases attention leading to speaking in a louder voice, (2) risk rises when large numbers of people are in a small space for a long time, and (3) sharing glasses and chopsticks increases the risk of infection [33]. 

We divided risky behaviors into two categories: “behaviors about socializing” and “behaviors about interactions”. In this study, “behaviors about socializing” is defined as talking closely for a long time with family and friends, sometimes with masks removed, and includes the items “dine out”, “drink at restaurants” and “go out” in this study, while “behaviors about interactions” is defined as exchanges mainly with strangers, and contains “not practicing safety precautions” and “travel”. We felt the need to divide risky behaviors into these two categories because “interaction” is more reducible, social structure-wise, with technologies such as Zoom conferencing and delivery services, and more controllable with strict enforcement of precautionary measures (i.e., mask-wearing and hand-washing). Studies show that aside from unfounded beliefs about the ineffectiveness of masks, about how coronavirus is a hoax, or about particular political ideologies, the vast majority of people cite “physical discomfort or negative effects” or “lack of mask-wearing culture” as their objections to safety precautions [34]. In other words, not practicing safety precautions allows people to save time, save the hassle of developing a new habit, have better breath, have a better experience, and experience more pleasure. Risky behaviors about interactions are more pleasure-seeking or orientated toward minimizing discomfort. Socializing with friends, however, is more complex. It carries enormous risks of transmitting the virus; on the other hand, it has been shown to be associated with less perceived stress, better mental health [35], more social networks, and more social support [36], especially during the pandemic [37]. People are generally more concerned when their social contact behaviors are restricted rather than interactions. 

Participants were asked about the frequencies of these risk behaviors (i.e., “rarely”, “a few times per month”, “1–2 days per week”, “3–4 days per week”, and “almost every day”). The lines for high-risk behaviors were set approximately at the 10th to 20th percentiles, where the majority of medical students decided not to take risks at that frequency. In this study, high-risk behaviors contained six or more travels in the past year, dining out for three or more days per week, drinking at restaurants for a few times or more per month, going out with friends for one or more days per week, and not practicing more than two accounts of the safety precautions listed above.

Considering the discrepancies in intentions and behaviors, we chose actual risky behaviors as our outcomes.

#### 2.3.3. Covariates

Covariates included age, gender, household income, and self-evaluation of COVID-19 infection risk. We included age, gender, and household income factors since risk preference is known to be associated with them [5]. For anonymous purposes, we did not include height, and instead of concrete age, we chose to separate age into three groups based on the status of admission: those who had the experience of going to another university, those who had gap years after graduating high school, and those who enrolled immediately after graduating high school. Those who enrolled immediately after graduating from high school were 21–22 years old. The majority of students who had gap years after graduating from high school were 22–23 years old, and two students who were 23–24 years old may or may not have participated in this survey. Those who had the experience of going to another university range from 22 to30 years old, with the majority being 24–25 years old. Household income was assessed in categories of “>JPY 10 million”, “JPY 8–10 million”, “JPY 6–8 million”, “JPY 4–6 million”, “JPY 2–4 million”, “<JPY 2 million”, and “do not know or do not want to answer”. 

Self-evaluation of COVID-19 infection risk was added to exclude the better-than-average effect. The survey included three questions to assess the better-than-average effect: self-confidence for not contracting the virus, for not transmitting the virus to their families, and for the condition not worsening even if contracted, compared to one’s peers. All three items were assessed on a scale of 1 (very unlikely) to 7 (very likely). 

#### 2.3.4. The Overconfidence Effect

We were cautious about the overconfidence effect in young people. The overconfidence effect is also known as the optimism bias or the better-than-average effect [38]. It is the tendency to rate one’s current abilities, attributes, or personality traits more favorably than the average peer, and is a prominent manifestation of self-evaluation bias [39,40]. Research has shown that risk perception is related to knowledge and information from various sources [41] and current health status [42]; thus, adjusting for the overconfidence effect is crucial to investigate the association of risk preference and risk behaviors in the long-term without the noise of short-term influences from media or events and health status.

### 2.4. Statistical Analysis

The association between risk preference and risk behaviors was analyzed by ordered logistic regression since the frequency of conducting risk behaviors was used as a categorical variable (i.e., “rarely”, “a few times per month”, “1–2 days per week”, “3–4 days per week”, and “almost every day”). In the crude model we analyzed the association between specific risk behaviors and three types of risk preferences using ordered logistic regression, which is a type of regression analysis used when the dependent variable is ordinal [43]. In this case, we sorted the frequency of risky behaviors into ordinal categories. We created two adjusted models. Model 1 includes the standard covariates that are commonly adjusted in risk preference studies: gender, age, and household income, which are demonstrated to be associated with risk preference in previous studies [20]. In model 2, we additionally adjusted for the overconfidence scale in order to obtain a clearer picture of how risk behaviors are affected by risk preferences without the noise of information asymmetry or personal overconfidence. This type of estimation used the maximum likelihood estimation technique by Stata. The significance level was set to 0.05. All analyses were conducted using Stata SE 15.0 (Stata Statistical Software, 2017, StataCorp LLC. StataCorp., College Station, TX, USA).

## 3. Results

The demographic of the participants is shown in Table 1. A total of 98 volunteers out of 108 fourth-year medical students (response rate 90.7%, 36.7% of women) completed the online survey. The proportions of those who enrolled right after graduation, enrolled after one or more gap years, and re-enrolled were 67.4%, 18.4%, and 14.3%, respectively. As for annual household income, 27.6% reported less than JPY 10 million, 45.9% reported over JPY 10 million, and 26.5% stated that it was not applicable (“do not know or do not want to answer”). High-risk behaviors, for which we set the bar at six or more travels in the past year, dining out for three or more days per week, drinking at restaurants for a few times or more per month, going out with friends for one or more days per week, and not practicing more than two accounts of safety precautions, were 10.2%, 9.2%, 19.4%, 23.5% and, 23.5%, respectively.

The correlations between the types of risk preferences are demonstrated in Table 2. The results were broadly consistent with previous findings. The weak correlation between general and hedonic preferences was consistent with previous research [5]. Monetary preference was not significantly correlated with the general risk preference, which was also supported by previous studies [11,44]. With a similar format but different context and process, monetary and hedonic preferences were also weakly correlated. 

The odds ratios (ORs) for each type of risk preference and high-risk behaviors are shown in Table 3. In the crude model, participants who showed risk-seeking inclinations in general and hedonic preferences were 4.41 and 5.87 times more likely to engage in high-risk behaviors, respectively (95% confidence interval (CI): 1.24–15.70 and 1.80–19.17). The association remained significant after adjustment for gender, age, and household income in model 1. We also found a significant association in model 2; risk-seeking in general and hedonic preferences were 4.04 and 6.58 times more likely, respectively, to engage in high-risk behaviors (95% CI: 1.05–15.50 and 1.86–23.28). Monetary preference was not significantly associated with high-risk behaviors in model 2 (OR: 0.78, 95% CI: 0.25–2.43) or in any model. 

Regarding specific risk behaviors (see Table 4 and Table 5), hedonic preference was significantly associated with all risk behaviors except for drinking at restaurants in all three models. In model 2, participants with a lower propensity to take risks, as measured by hedonic preference, were less likely to “dine out” (OR: 2.78, 95% CI: 1.13–6.85), “go out” (OR: 4.35, 95% CI: 1.65–11.46), to be “not practicing safety precautions” (OR: 2.79, 95% CI: 1.11–7.04), and to “travel” (OR: 4.36, 95% CI: 1.42–13.44). Risk-seeking measured by the general preference was significantly associated with two accounts of risky behaviors, that is, “dining out” and “going out” (OR: 4.82 and 6.48, 95% CI: 1.66–14.00 and 2.07–20.24 in model 2, respectively). Risk-seeking in monetary preference showed no significant association with any risk behavior in any model.

## 4. Discussion

In this study, we found that for high-risk behaviors in general, general risk preference and hedonic preferences were 4.04 and 6.58 times more likely, respectively, to engage in high-risk behaviors when controlling for knowledge and the overconfidence effect. Monetary preference was not significantly associated with high-risk behaviors.

To the best of our knowledge, this is the first study to show the association between risk preference and risk behaviors in Japan, the first to include the overconfidence effect in COVID-19 risk behavior studies, and the first attempt to account for the specific context and decision-making process of COVID-19 in risk preference studies. Our research adds to the current literature on the association of risky behaviors and risk preferences while controlling for knowledge and the overconfidence effect and provides a novel method with the specific COVID-19 context and decision-making process.

Consistent with previous studies [18,19], the established approach of the general risk preference question still functions well as a predictor of risky behaviors. The small effect size of monetary preference is consistent with previous studies [11], in which its poor ability to predict real-life decision-making was confirmed. The hedonic preference is a novel concept specifically designed for accommodating the context and the decision-making process of the COVID-19 pandemic. It is measured by the risk-for-pleasure-seeking preference question. The feasibility of this novel measurement is demonstrated by its stronger association with risky behaviors. It also highlights the importance of context- and process-based assessment tools.

When comparing hedonic and monetary preferences, the format is essentially the same, yet the addition of the specific COVID-19 context and process caused differences of significant association in the hedonic preference and none in the monetary preference. 

Regarding specific risky behaviors, the general risk preference is 4.82 and 6.48 times more likely to “dine out” and “go out with friends” (socializing), respectively; and the hedonic preference is 2.78, 2.79, and 4.36 times more likely to “dine out”, “not practice precautionary measures”, and “travel” (interaction), respectively. On the other hand, monetary preference is not significantly associated with any risky behaviors. 

The reason that risky behaviors about socializing are more likely to occur to people with risk-seeking tendency measured by the general preference may be due to the intentionally vague context of this measurement. One possible explanation is that the general preference question essentially inquires about what first comes to mind when people are asked about risk-taking, and of the risky behaviors in the COVID-19 pandemic, socializing behaviors are often more memorable and therefore come up more often when asked vaguely. There are still many unexplored areas in the field of general risk preference research. Some researchers favor the deliberate black box structure [5], while others try to ascertain the mechanism behind it through various approaches, including behavioral science, neuroscience, and psychology [45]. In any case, the practical application is the strength of the general preference question, confirmed by our study.

On the other hand, the risk-seeking tendency shown in the hedonic preference predicts a higher likelihood of risky behaviors about interactions (i.e., “not practicing safety precautions” and “travel”). Risky behaviors about interactions are more pleasure-seeking or more orientated toward minimizing discomfort since the main reason for such risky behaviors is to avoid “physical discomfort or negative effects” or “lack of mask-wearing culture” [34], meaning to save time, save the trouble of developing a new habit, and have a better experience. “Socializing” behaviors also have pleasure-seeking elements because people do not have to endure loneliness during the pandemic, which explains why hedonic preference has some predictive power in socializing. Overall, the association between certain risky behaviors under the pandemic and the hedonic preference can be reasonably explained. The incapability of the monetary preference to predict specific risky behaviors is consistent with previous literature [11]. With the proper differentiation of people whose risk preferences are elicited by general and hedonic questions, we can predict which group of people are more likely to be risk-seeking in which types of behaviors. 

### 4.1. Implication

Our study has policy implications. Because risk preferences are considered to be relatively stable across the course of a person’s life, with little influence from short-term effects on mental health or mood [46,47,48], and to remain stable during the COVID-19 pandemic [49,50], they may serve as reliable long-term predictors of future risky behaviors. Based on our findings, further large-scale studies may effectively predict, differentiate, and track high-risk groups who are more likely to engage in risky behaviors during the current pandemic or even future pandemics. While the evidence on the contribution of each risky behavior to the spread of the virus is insufficient, with the proper use of general and hedonic preferences, we can better differentiate people who are inclined to certain types of risky behaviors and target them with more concrete incentives or restrictions, which would benefit more effective policy-making. This may also apply to policy-making in other pandemics and epidemics with similar transmission patterns.

Another issue that cannot be ignored in policy-making is the cost of mitigation measures. “Interactions” are more scalable, in the sense that strict enforcement of precautionary measures (i.e., mask-wearing and hand-washing) would not typically cause severe damage to the economy and public health. However, restricting “socializing” has more risks, as it can lead to more perceived stress and mental health problems [35], fewer social networks, and less support [36], especially during the pandemic [37]. With the results of our study, we can estimate the numbers of people who will be affected by each mitigation measure and weigh whether the benefits triumph over the harms.

We have also taken steps to ensure that our measurements are culturally inclusive. Cross-national studies can face many obstacles; for instance, the DOSPERT scale introduced in Japan performed poorly among the Japanese population [51]. Six of the questions related to sexual violence and drugs cannot even be surveyed due to Japanese laws and regulations. In our study, we explicitly avoid any controversial keywords or moral judgments and choose a situation that is accommodating to most major cultures. Given that cross-cultural studies are crucial to international negotiations—opening borders, vaccine passports, global health cooperation—a broader scope of future studies on other nations is warranted.

### 4.2. Limitation

This study has several limitations. First, since the current study is cross-sectional, we cannot determine the causality of the association. Although previous studies have established that risk preferences are stable across time, even under the influence of the pandemic [46,47,48,49,50], we cannot categorically deny the possibility of bi-directional association. Second, the sample size of participants is relatively small, restricted by the size of a medical school. Further study of the population on a larger scale may reveal more details. Third, the participants of this study are medical students. Most of them have similar backgrounds (i.e., parents’ socioeconomic status, education, knowledge of medical issues, childhood environment, ethnicity) that may impact their risk preferences [52]. In light of this criticism, a broader range of participants is warranted in extensive surveys. Fourth, we were not able to examine the criterion-related validity of our scale because the COVID-19 pandemic is an unprecedented global pandemic that changed the definition of risky behaviors, and no previous research has addressed this specific context. 

## 5. Conclusions

As hypothesized, we found that young people in Japan who showed risk-seeking inclinations in hedonic preference were approximately 6.5 times more likely, and those in general preference were around 4 times more likely, to engage in high-risk behaviors during the COVID-19 pandemic, controlling for knowledge and the overconfidence effect. Our study demonstrates that even when awareness, knowledge, and the overconfidence effect are removed from the equation, risk preference alone is significantly associated with risk behaviors. In addition, the general risk preference is 4.82 and 6.48 times more likely to “dine out” and “go out with friends”, respectively; and the hedonic preference is 2.78, 2.79, and 4.36 times more likely to “dine out”, “not practice precautionary measures” and “travel”, respectively. The evidence strongly supports the feasibility of the novel measurement of hedonic preference for future surveys.

## Figures and Tables

**Table 1:** Demographics.

			Total (*n* = 98)	
			*n*	%
Characteristics	Sex	Male	62	63.3
		Female	36	36.7
	Age	Have experience of going to another university	14	14.3
		Had gap years after graduating high school	18	18.4
		Enrolled immediately after graduating high school	66	67.4
	Household income	< JPY ten million	27	27.6
		≧JPY ten million	45	45.9
		Do not know/do not want to answer	26	26.5
Risk behaviors	Travels (in the past year)	0–5	88	89.8
		6 or more times	10	10.2
	Dining out	Less than 3 days per week	89	90.8
		3 or more days per week	9	9.2
	Drinking at restaurants	Rarely	79	80.6
		A few times or more per month	19	19.4
	Going out (with friends)	Less than 1 day per week	75	76.5
		1 or more days per week	23	23.5
	Accounts of safety precautions regularly practiced (out of wearing masks, washing hands, taking one’s temperature, and avoiding crowds)	1–2	16	16.3
		3–4	82	83.7
	Any high-risk behaviors		43	43.9

**Table 2:** Spearman correlation coefficients between types of risk preferences.

	Risk-Averse (*n*, %)	Risk-Seeking (*n*, %)	(1)	(2)	(3)
(1) General preference	80 (81.6%)	18 (18.4%)	1.00		
(2) Monetary preference	71 (72.5%)	27 (27.5%)	0.14	1.00	
(3) Hedonic preference	75 (76.5%)	23 (23.5%)	**0.30**	**0.30**	1.00

Note: **Bold**: *p* < 0.05.

**Table 3:** Association between types of risk preference and high-risk behaviors.

		Crude		Model 1		Model 2	
		OR (95% CI)	*p*-Value	OR (95% CI)	*p*-Value	OR (95% CI)	*p*-Value
General preference	Risk-averse	ref		ref		ref	
	Risk-seeking	**4.41 (1.24–15.70)**	**0.022**	**3.89 (1.03–14.63)**	**0.045**	**4.04 (1.05–15.50)**	**0.042**
Monetary preference	Risk-averse	ref		ref		ref	
	Risk-seeking	0.89 (0.31–2.60)	0.835	0.86 (0.28–2.59)	0.783	0.78 (0.25–2.43)	0.664
Hedonic preference	Risk-averse	ref		ref		ref	
	Risk-seeking	**5.87 (1.80–19.17)**	**0.003**	**6.84 (1.97–23.80)**	**0.003**	**6.58 (1.86–23.28)**	**0.003**

Note: **Bold**: *p* < 0.05. No covariates were adjusted in the crude model; gender, age, and household income were adjusted in model 1; and gender, age, household income, and self-evaluation of COVID infection risk were adjusted in model 2. General preference is assessed by the self-reported general risk preference question. Monetary preference is assessed by the expected utility-based lottery choice methods. Hedonic preference is assessed by the risk-for-pleasure-seeking preference question.

**Table 4:** Association between types of risk preferences and risky behaviors about socializing.

			Crude		Model 1		Model 2	
			OR (95% CI)	*p*-Value	OR (95% CI)	*p*-Value	OR (95% CI)	*p*-Value
Dining out	General preference	Risk-averse	ref		ref		ref	
		Risk-seeking	**4.73 (1.74–12.85)**	**0.002**	**4.82 (1.67–13.94)**	**0.004**	**4.82 (1.66–14.00)**	**0.004**
	Monetary preference	Risk-averse	ref		ref		ref	
		Risk-seeking	1.06 (0.47–2.41)	0.888	1.03 (0.45–2.37)	0.947	0.95 (0.41–2.19)	0.897
	Hedonic preference	Risk-averse	ref		ref		ref	
		Risk-seeking	**3.11 (1.30–7.45)**	**0.011**	**2.92 (1.19–7.18)**	**0.020**	**2.78 (1.13–6.85)**	**0.026**
Drinking at restaurants	General preference	Risk-averse	ref		ref		ref	
		Risk-seeking	0.84 (0.22–3.28)	0.806	0.87 (0.20–3.75)	0.848	0.85 (0.19–3.77)	0.829
	Monetary preference	Risk-averse	ref		ref		ref	
		Risk-seeking	2.41 (0.85–6.84)	0.099	2.80 (0.93–8.41)	0.066	2.48 (0.81–7.59)	0.110
	Hedonic preference	Risk-averse	ref		ref		ref	
		Risk-seeking	1.22 (0.39–3.84)	0.731	1.39 (0.41–4.68)	0.595	1.15 (0.33–4.03)	0.832
Going out	General preference	Risk-averse	ref		ref		ref	
		Risk-seeking	**5.98 (2.07–17.27)**	**0.001**	**5.82 (1.89–17.92)**	**0.002**	**6.48 (2.07–20.24)**	**0.001**
	Monetary preference	Risk-averse	ref		ref		ref	
		Risk-seeking	1.95 (0.84–4.53)	0.120	1.78 (0.75–4.19)	0.189	1.45 (0.62–3.46)	0.396
	Hedonic preference	Risk-averse	ref		ref		ref	
		Risk-seeking	**5.48 (2.13–14.06)**	**<0.001**	**5.05 (1.94–13.15)**	**0.001**	**4.35 (1.65–11.46)**	**0.003**

Note: **Bold**: *p* < 0.05. No covariates were adjusted in the crude model; gender, age and household income were adjusted in model 1; and gender, age, household income, and self-evaluation of COVID infection risk were adjusted in model 2.

**Table 5:** Association between types of risk preferences and risky behaviors about interactions.

			Crude		Model 1		Model 2	
			OR (95% CI)	*p*-Value	OR (95% CI)	*p*-Value	OR (95% CI)	*p*-Value
Not practicing safety precautions	General preference	Risk-averse	ref		ref		ref	
		Risk-seeking	1.54 (0.57–4.16)	0.400	1.82 (0.63–5.28)	0.270	1.81 (0.62–5.25)	0.277
	Monetary preference	Risk-averse	ref		ref		ref	
		Risk-seeking	0.70 (0.30–1.66)	0.420	0.72 (0.30–1.72)	0.459	0.66 (0.27–1.62)	0.368
	Hedonic preference	Risk-averse	ref		ref		ref	
		Risk-seeking	**2.52 (1.05–6.04)**	**0.038**	**2.91 (1.17–7.27)**	**0.022**	**2.79 (1.11–7.04)**	**0.030**
Travel	General preference	Risk-averse	ref		ref		ref	
		Risk-seeking	2.61 (0.89–7.68)	0.082	2.79 (0.88–8.89)	0.083	2.74 (0.86–8.75)	0.089
	Monetary preference	Risk-averse	ref		ref		ref	
		Risk-seeking	2.27 (0.845–6.08)	0.104	2.43 (0.88–6.72)	0.086	2.37 (0.85–6.59)	0.100
	Hedonic preference	Risk-averse	ref		ref		ref	
		Risk-seeking	**4.52 (1.55–13.18)**	**0.006**	**4.42 (1.46–13.38)**	**0.008**	**4.36 (1.42–13.44)**	**0.010**

Note: **Bold**: *p* < 0.05. No covariates were adjusted in the crude model; gender, age and household income were adjusted in model 1; and gender, age, household income, and self-evaluation of COVID infection risk were adjusted in model 2.

## Data Availability

All datasets generated for this study are included in the article/Supplementary Material.

## Supplementary Materials

The following supporting information can be downloaded at: https://www.mdpi.com/article/10.3390/ijerph20126090/s1, Table S1: Association between types of risk preferences and specific risk behaviors; Table S2: Demographic of types of risk preferences; Questionnaire S3: Questionnaire used in this study.
