# Supplementary material for: Hedonic Risk Preference Associated with High-Risk Behaviors under COVID-19 Pandemic among Medical Students in Japan
Source: Int J Environ Res Public Health. 2023 Jun 9;20(12):6090. doi: 10.3390/ijerph20126090 (PMC10298028; doi:10.3390/ijerph20126090)
Supplement: Supplementary file 1 [file ijerph-20-06090-s001.zip › ijerph-2395405-supplementary.pdf]

Hedonic risk preference associated with high-risk behaviors under COVID-19 pandemic among medical students in Japan

Zechen Zeng[1], Nobutoshi Nawa[2], Chie Hirama[1], Takeo Fujiwara[1*]

[1] Department of Global Health Promotion, Tokyo Medical and Dental University, 1-5-45 Yushima, Bunkyo-ku, Tokyo, Japan.

[2] Department of Medical Education Research and Development, Tokyo Medical and Dental University, Tokyo, Japan

Corresponding author:

Takeo Fujiwara, MD, PhD, MPH

Department of Global Health Promotion, Tokyo Medical and Dental University, 1-5-45 Yushima, Bunkyo-ku, Tokyo 113-8519, Japan.

E-mail: fujiwara.hlth@tmd.ac.jp

Tel: +81-3-5803-5187 Fax: +81-3-5803-5190

Supplementary Table S1. Association between types of risk preferences and specific risk behaviors

Method	Simple or Complex	Quantitative or Qualitative	Reviewed by	Summary
General preference	Simple	Qualitative	Charness et al. (2013),[1] Lonnqvist et al. (2015) [2]	Predicted behavior across all domains;[20] questionnaire data predicted actual risk-taking behavior.[2]
Monetary preference	Simple ~ Complex [1]	Quantitative	Charness et al. (2013),[1] Lonnqvist et al. (2015) [2]	Had little predictive power; [20] did not predict actual risk-taking behavior.[2]
Hedonic preference	Simple	Quantitative	**N/A**	Associated with high-risk behaviors specifically during the COVID-19 pandemic.
DOSPERT	Simple	Qualitative	Charness et al. (2013),[1] Lonnqvist et al. (2015) [2]	Best predictors of self-reported risky behavior in respective domain; [20] questionnaire data predicted actual risk-taking behavior.[2]
Incentivized economic experiments	Complex	Quantitative	Charness et al. (2013),[1]	Significant correlations between risk preferences elicited using monetary and experiments methods.[3]

Note: 1. Single question is simple and multiple-choice form (i.e., the Multiple Price List) is complex.

Supplementary Table S2. Demographic of types of risk preferences

	Proportion of general preference		Proportion of monetary preference		Proportion of hedonic preference	
	Risk-seeking 80 (81.6%)	Risk-seeking 18 (18.4%)	Risk-seeking 71 (72.5%)	Risk-averse 27 (27.6%)	Risk-seeking 75 (76.5%)	Risk-averse 23 (23.5%)
Age						
Have experience of going to another universities	12 (15.0%)	2 (11.1%)	8 (11.3%)	6 (22.2%)	11 (14.7%)	3 (13.0%)
Had gap years after graduating high school	17 (21.3%)	1 (5.6%)	14 (19.7%)	4 (14.8%)	15 (20.0%)	3 (13.0%)
Enrolled immediately after graduating high school	51 (77.3%)	15 (22.7%)	49 (69.0%)	17 (63.0%)	49 (65.3%)	17 (73.9%)
Gender						
female	34 (42.5%)	2 (11.1%)	28 (39.4%)	8 (29.6%)	31 (41.3%)	5 (21.7%)
male	46 (57.5%)	16 (88.9%)	43 (60.6%)	19 (70.4%)	44 (58.7%)	18 (78.3%)
Travels (in the past year)						
0	24 (30.0%)	2 (11.1%)	19 (26.8%)	7 (25.9%)	24 (32.0%)	2 (8.7%)
1–5 times	49 (61.3%)	13 (72.2%)	49 (69.0%)	13 (48.2%)	46 (61.3%)	16 (69.6%)
6–10 times	4 (5.0%)	2 (11.1%)	3 (4.2%)	3 (11.1%)	4 (5.3%)	2 (8.7%)
11–15 times	2 (2.5%)	1 (5.6%)	0 (0.0%)	3 (11.1%)	1 (1.3%)	2 (8.7%)
16 times and over	1 (1.25%)	0 (0.0%)	0 (0.0%)	0 (0.0%)	0 (0.0%)	1 (4.4%)
Dining out						
Rarely	24 (30.0%)	3 (16.7%)	19 (26.8%)	8 (29.6%)	24 (32.0%)	3 (13.0%)
A few times per month	34 (42.5%)	2 (11.1%)	27 (38.0%)	9 (33.3%)	29 (38.7%)	7 (30.4%)
1–2 days per week	17 (21.3%)	9 (50.0%)	20 (28.2%)	6 (22.2%)	17 (22.7%)	9 (39.1%)
3–4 days per week	2 (2.5%)	3 (16.7%)	2 (2.8%)	3 (11.1%)	3 (4.0%)	2 (8.7%)
Almost everyday	3 (3.8%)	1 (5.6%)	3 (4.2%)	1 (3.7%)	2 (2.7%)	2 (8.7%)
Drinking at restaurants						
Rarely	64 (80.0%)	15 (83.3%)	60 (84.5%)	19 (70.4%)	61 (81.3%)	18 (78.3%)
A few times per month	14 (17.5%)	2 (11.1%)	10 (14.1%)	6 (22.2%)	12 (16.0%)	4 (17.4%)
1–2 days per week	2 (2.5%)	0 (0.0%)	1 (1.4%)	1 (3.7%)	1 (1.3%)	1 (4.4%)
3–4 days per week	0 (0.0%)	1 (5.6%)	0 (0%)	1 (3.7%)	1 (1.3%)	0 (0%)
Almost everyday	0 (0.0%)	0 (0.0%)	0 (0%)	0 (0%)	0 (0%)	0 (0%)
Going out (with friends)						
Rarely	28 (35.0%)	3 (16.7%)	24 (33.8%)	7 (25.9%)	28 (37.3%)	3 (13.0%)
A few times per month	39 (48.8%)	5 (27.8%)	34 (47.9%)	10 (37.0%)	36 (48.0%)	8 (34.8%)
1–2 days per week	12 (15.0%)	6 (33.3%)	10 (14.1%)	8 (29.6%)	9 (12.0%)	9 (39.1%)
3–4 days per week	1 (1.3%)	4 (22.2%)	3 (4.2%)	2 (7.4%)	2 (2.7%)	3 (13.0%)
Almost everyday	0 (0.0%)	0 (0.0%)	0 (0%)	0 (0%)	0 (0%)	0 (0%)
Safety precautions						
Wearing mask	80 (100%)	18 (100%)	71 (100%)	27 (100%)	75 (100%)	23 (100%)
Washing hand	75 (93.8%)	15 (83.3%)	64 (90.1%)	26 (96.3%)	69 (92%)	21 (91.3%)
Measuring temperature	52 (65.0%)	11 (61.1%)	43 (60.6%)	20 (74.1%)	52 (69.3%)	11 (47.8%)
Avoiding crowd	65 (81.3%)	13 (50.0%)	57 (80.3%)	21 (77.8%)	62 (82.7%)	16 (69.6%)
Accounts of safety precautions Regularly practicing						
0	0 (0.0%)	0 (0.0%)	0 (0.0%)	0 (0.0%)	0 (0.0%)	0 (0.0%)
1	1 (1.3%)	1 (5.6%)	2 (2.8%)	0 (0.0%)	2 (2.7%)	0 (0.0%)
2	10 (12.5%)	4 (22.2%)	11 (15.5%)	3 (11.1%)	8 (10.7%)	6 (26.1%)
3	25 (31.3%)	4 (22.2%)	21 (29.6%)	8 (29.6%)	20 (26.7%)	9 (39.1%)
4	44 (55.0%)	9 (50.0%)	37 (52.1%)	16 (59.3%)	45 (60.0%)	8 (24.8%)

Questionnaire S3. Questionnaire used in this study.

1) What is your gender?
   1. Male 2. Female 3. Others

2) Choose your enrollment status. (Having experience of going to another university includes transfer students and those who applied to the university while already enrolled in another)
   1. Enrolled immediately after graduating high school
   2. Have blank years after graduating high school
   3. Have experience of going to another university

3) How likely do you think you could be infected with coronavirus compared to your average peers?

Very unlikely	1	2	3	4	5	6	7	Very likely

4) If you were infected with coronavirus, compared to your average peers, how likely do you think it is that you would pass it on to your family and friends?

Very unlikely	1	2	3	4	5	6	7	Very likely

5) If you were infected with coronavirus, compared to your average peers, how likely do you think it is that condition could be severe or left with prognostic symptoms?

Very unlikely	1	2	3	4	5	6	7	Very likely

6) During the past six months, on average, how often have you been dining out?
   1. Rarely 2. A few times per month 3. 1~2 days per week 4. 3~4 days per week 5. Almost everyday

7) During the past six months, on average, how often have you been going out with friends?
   1. Rarely 2. A few times per month 3. 1~2 days per week 4. 3~4 days per week 5. Almost everyday

8) During the past six months, on average, how often have you been drinking at restaurants?
   1. Rarely 2. A few times per month 3. 1~2 days per week 4. 3~4 days per week 5. Almost everyday

9) During the past year, how often have you been traveling?
   1. 0 2. 1~5 times 3. 6~10 times 4. 10~15 times 5. Over 20 times

10) Select all the infection precaution measures that you have been regularly practicing during the COVID-19 pandemic.
   1. Wearing masks 2. A Washing hands 3. Measuring temperature 4. Avoiding crowd

11) Choose the range of your annual household income as far as you know.
   1. 0–2 million yen

2. 2–4 million yen
3. 4–6 million yen
4. 6–8 million yen
5. 8–10 million yen
6. Over 10 million yen

7. Don't know/don't want to answer

12) Do you live with your parents?
1. Yes 2. No

13) Do you consider yourself a person who would take risks in general?
1. Yes 2. No

14) Suppose you won the lottery, and you can choose from the four rewards below. Which one would you choose?
1. Win 40,000 yen with a 100% chance

2. Win 50,000 yen with an 80% chance, but 20% of nothing

3. Win 80,000 yen with a 50% chance, but 50% of nothing

4. Win 100,000 yen with a 50% chance, but 50% of nothing

15) Please select the reason for your choice.
1. I want money for sure. 2. Given the chance, I want more money 3. The expectation is the

highest. 4. I don't need money/I don't care. 5. Others:

16) Suppose you were a famous YouTuber, and you love drinking with large groups of people. You are often invited to large drinking parties by your friends. However, there is a risk that the public will find out about it, and if they do, you will be suspended for a while and will not be able to go out drinking. You have been vaccinated and do not consider yourself at risk of infection. The only thing you care about in this world is the number of parties you can go to. What would you choose in the following situation?
1. Party 4 times a year with a 100% chance of not getting caught

2. Given a chance, I want more money

3. The expectation is the highest.

4. I don't need money/I don't care.

5. Others:

17) Please select the reason for your choice.
    1. I want parties for sure.

    2. Given the chance, I want more parties

    3. The expectation is the highest.

    4. I don't like parties/I don't care.

    5. Others:

1. Charness G, Gneezy U, Imas A. Experimental methods: Eliciting risk preferences. *Journal of Economic Behavior & Organization*. 2013;87:43-51.
2. Lönnqvist J-E, Verkasalo M, Walkowitz G, Wichardt PC. Measuring individual risk attitudes in the lab: Task or ask? An empirical comparison. *Journal of Economic Behavior & Organization*. 2015;119:254-266.
3. Dohmen T, Falk A, Huffman D, Sunde U, Schupp J, Wagner GG. Individual risk attitudes: Measurement, determinants, and behavioral consequences. *Journal of the european economic association*. 2011;9(3):522-550.
